# Geometric Calibration and Accuracy Verification of the GF-3 Satellite

**DOI:** 10.3390/s17091977

**Published:** 2017-08-29

**Authors:** Ruishan Zhao, Guo Zhang, Mingjun Deng, Kai Xu, Fengcheng Guo

**Affiliations:** 1 School of Geomatics, Liaoning Technical University, Fuxin 123000, China; zhaoruishan333@163.com; 2 State Key Laboratory of Information Engineering in Surveying Mapping and Remote Sensing, Wuhan University, Wuhan 430079, China; 2015206190081@whu.edu.cn (K.X.); fch_guo@163.com (F.G.); 3 School of Remote Sensing and Information Engineering, Wuhan University, Wuhan 430079, China; dmj2008@whu.edu.cn

**Keywords:** SAR, GF-3 satellite, atmospheric propagation delay correction, geometric positioning accuracy, ground control points

## Abstract

The GF-3 satellite is the first multi-polarization synthetic aperture radar (SAR) imaging satellite in China, which operates in the C band with a resolution of 1 m. Although the SAR satellite system was geometrically calibrated during the in-orbit commissioning phase, there are still some system errors that affect its geometric positioning accuracy. In this study, these errors are classified into three categories: fixed system error, time-varying system error, and random error. Using a multimode hybrid geometric calibration of spaceborne SAR, and considering the atmospheric propagation delay, all system errors can be effectively corrected through high-precision ground control points and global atmospheric reference data. The geometric calibration experiments and accuracy evaluation for the GF-3 satellite are performed using ground control data from several regions. The experimental results show that the residual system errors of the GF-3 SAR satellite have been effectively eliminated, and the geometric positioning accuracy can be better than 3 m.

## 1. Introduction

The GF-3 satellite is the first multi-polarization synthetic aperture radar (SAR) imaging satellite in China, which operates in the C band with a resolution of 1 m. It is also the first low earth orbit (LEO) remote sensing satellite in China with a design life of 8 years, and was launched on 10 August 2016. It has 12 imaging modes, including the traditional StripMap mode and the ScanSAR mode, as well as a wave imaging mode for marine applications and a global observation imaging mode. In fact, GF-3 has the largest number of imaging modes of any SAR satellite in the world. It can provide data support services with long-term stability; for example, global land and ocean resources can be monitored during all weather conditions and at any time, and microwave remote sensing data of different application modes can be obtained with high effectiveness. Furthermore, in the future, new techniques can enable the monitoring of the marine environment, marine licenses, and conservation interests; the monitoring and assessment of natural disasters; the monitoring of water conservation facilities; the evaluation and management of water resources; meteorological research; and other services.

Geometric positioning accuracy is one of the important technical indexes of SAR satellites and a key factor affecting the processing and application of SAR satellite image data. As a result, the geometric positioning capability of advanced spaceborne SAR systems is being continuously improved. The European ERS satellite is the oldest SAR satellite, and its plane positioning accuracy can reach 20 m [1]. The COSMO-SKYMED satellite of Italy can achieve a positioning accuracy of 15 m, and the geometric positioning accuracy of ALOS in Japan, RadarSat-2 in Canada, TerraSAR-X in Germany, and SENTINEL-1A of the European Space Agency (ESA) can exceed 10 m [2,3,4,5,6,7,8].

Although the SAR system of the GF-3 satellite was geometrically calibrated during the in-orbit commissioning phase, some system errors still exist that affect its geometric positioning accuracy. In this study, we classify these errors into three categories; namely, fixed system error, time-varying system error, and random error. We then correct for these system errors, thereby improving the geometric positioning accuracy of the GF-3 SAR satellite, by performing multimode hybrid geometric calibration of the spaceborne SAR, considering the atmospheric propagation delay, and using high-precision ground control points (GCPs) and global atmospheric reference data. The geometric calibration and accuracy evaluation of GF-3 are performed using ground control data from several different regions.

In this paper, Section 2 describes the GF-3 SAR system description. Section 3 analyzes the three types of spaceborne SAR geometric positioning errors, and Section 4 describes the geometric calibration and accuracy verification methods used in this research. The calibration and verification results for the GF-3 satellite are discussed in Section 5, and our conclusions are presented in Section 6.

## 2. GF-3 SAR System Description

The GF-3 satellite is a high-resolution, fully polarimetric SAR satellite, with 12 imaging modes. It operates on the solar synchronous orbit at a height of 755 km. The weight of the entire satellite is 2779 kg. The antenna type is a wave-guide slot phased array with an area of 15 m × 1.5 m. The design life of the satellite is 8 years. Details of the GF-3 satellite’s imaging modes are shown in Table 1.

Dual frequency Global Position System (GPS) receivers are used to provide orbital parameters in the WGS-84 coordinate system on the GF-3 satellite. According to real-time orbit data downloaded from GPS receivers installed on the satellite, the position accuracy is better than 10 m. Based on precise satellite orbit determination post-processing, the position accuracy can even achieve 5 cm [9].

To obtain a zero Doppler center frequency, two-dimensional yaw steering technology is used on the GF-3 satellite to eliminate effects induced by the rotation of the Earth, the oblateness of the Earth, the oblateness of the satellite orbit, etc. [10]. However, due to the presence of attitude control errors, there is still a small amount of Doppler frequency error generated in the satellite image. Doppler parameter information is provided in the auxiliary parameters file (*.meta.xml) of the level 1 product. The Doppler center frequency $f_{D}$ can be calculated using its fourth order of Tailor expansion in the vicinity of the current pixel’s fast time:

(1) $$f_{D}=a_{0}+a_{1}\left(t_{sr}-t_{0}\right)+a_{2}{\left(t_{sr}-t_{0}\right)}^{2}+a_{3}{\left(t_{sr}-t_{0}\right)}^{3}+a_{4}{\left(t_{sr}-t_{0}\right)}^{4}$$

where $a_{0}$, $a_{1}$, $a_{2}$, $a_{3}$, and $a_{4}$ are the five parameters provided in the auxiliary parameters file; $t_{sr}$ is the fast time of the current pixel; and $t_{0}$ is the fast time of the pixel in the near range. It should be noted that the calculated Doppler center frequency is the absolute value, and a minus sign should be added in positioning applications.

In addition, rational polynomial coefficient (RPC) parameters are provided in the level 1 product of the GF-3 satellite to facilitate subsequent user applications.

## 3. Geometric Positioning Error Analysis for Spaceborne SAR

The geometric positioning accuracy of spaceborne SAR is mainly affected by sensor instability, platform instability, signal propagation delay, terrain height, and processor-induced errors [9]. Regarding error characteristics, those that can affect the positioning accuracy of spaceborne SAR can be classified into fixed system errors, time-varying system errors, and random errors, described in the following subsections.

### 3.1. Fixed System Error

The ranging signals of the SAR system mainly depend on precise time measurement, including fast time (range direction) and slow time (azimuth direction). The two-dimensional time error is mainly affected by the SAR system time delay error and the azimuth time synchronization error, and is the main error source for the geometric positioning of spaceborne SAR.

The SAR system time delay error is mainly caused by the radar signal passing through each component of the signal channel. The pulse-width and bandwidth of the radar signal are the major factors contributing to the SAR system time delay [11]. During SAR satellite operation, the SAR system time delay errors of different pulse-width and bandwidth remain constant. The azimuth time synchronization error is mainly caused by the error of the time control unit of the system equipment. For the same spaceborne SAR system, the error is relatively stable and not affected by imaging modes and other factors. As a result, they are classified as fixed system errors.

### 3.2. Time-Varying System Error

Some of the error sources that affect geometric positioning accuracy vary with time. These mainly include the atmospheric propagation delay error and the error caused by imaging processing. The radar signal is affected by bidirectional atmospheric delay in the propagation path. The atmospheric propagation delay of radar signals is mainly related to atmospheric pressure intensity, temperature, water vapor content, ionospheric electron density, and the emission frequency of radar signals. Therefore, the atmospheric propagation delay error is a systematic error related to the incident angle of the radar beam and the imaging time of the SAR image.

Due to the different imaging times and scenes, the error caused by imaging processing also differs for each SAR image. The error of the Doppler center frequency will lead to a geometric positioning error in the azimuth direction. However, if the Doppler center frequency used in the positioning process is the same as that in the imaging process, no positioning error will be generated [9]. In addition, during the imaging processing of each SAR satellite image, it is assumed that the SAR system is stationary as it transmits and receives the same radar pulse signal, and the current moment is taken as the imaging time. However, the SAR satellite is in constant motion during the transmission and reception of the pulse signal, i.e., SAR satellite positions differ between transmission and reception times. Thus, the geometric positioning error in the azimuth direction is caused by an inconsistency in the time reference and is different for each SAR image.

### 3.3. Random Error

In general, it is difficult to effectively eliminate random error by ground treatment methods. Therefore, random error is the main factor affecting the theoretical limit of geometric localization accuracy in the SAR system. The random errors that affect geometry positioning accuracy are predominantly satellite position error, SAR system delay random error, SAR antenna dispersion error, control point error, and atmospheric propagation delay correction model error.

## 4. Geometric Calibration and Accuracy Verification of Spaceborne SAR

According to the relationship between fast time and slow time, we can see that [11]:

(2) $$\{\begin{array}{c} t_{f}=\left(t_{f0}+t_{delay}+\Delta t_{f}\right)+\frac{x}{f_{s}}\\ t_{s}=\left(t_{s0}+\Delta t_{s}\right)+\frac{y}{f_{p}} \end{array},x\in \left[0,width-1\right],y\in \left[0,hetght-1\right]$$

where *t_f_* and *t_s_* represent fast time in the range direction and slow time in the azimuth direction, respectively; *t_f_*_0_ and *t_s_*_0_ are the measured starting time values in the range and azimuth directions, respectively; *t_delay_* is the atmospheric propagation delay time; Δ*t_f_* and Δ*t_s_* are the system delay time errors; *f_s_* is the sampling frequency; *f_p_* is the pulse repetition frequency (PRF); x and y are pixel coordinates; and *width* and *height* describe the image size in the range and azimuth directions, respectively.

The propagation delay of the radar signal is mainly related to local atmospheric pressure, temperature, water vapor content, ionospheric electron density, and radar signal frequency, and can be divided into two main parts: that in the neutral atmosphere and that in the ionosphere. The atmospheric analysis model of the American National Center for Environmental Prediction (NCEP) was used to calculate the propagation delay correction of the radar signal in the neutral atmosphere, and Global Ionosphere Maps (GIM) in the Ionosphere Exchange (IONEX) format, provided every day by the European Centre for Orbit Determination (CODE), were used to calculate the propagation delay correction of the radar signal in the ionosphere.

The starting time of the satellite record is the time the radar signal is received. Imaging processing of the GF-3 satellite has an impact on the starting time in the azimuth direction. The approximate equivalent SAR imaging time is the intermediate time between the transmitting and receiving times [12]. Therefore, it should be corrected using:

(3) $$t_{s0}=t_{s0}^{\prime }-\frac{N/f_{prf}+t_{sample\_delay}}{2}$$

where $t_{s0}^{\prime }$ is the echo receiving time recorded on the satellite, *N* is the number of times the radar signal is transmitted from the transmitter to the receiver, and *t_sample_delay_* is the sample time delay in the satellite record.

The Range Doppler (RD) model is a rigorous imaging geometry model for spaceborne SAR that establishes a close relationship between the object coordinate and the image coordinate [11,13]:

(4) $$\left\{ \begin{array}{l} R=\sqrt{{\left(X_{t}-X_{s}\right)}^{2}+{\left(Y_{t}-Y_{s}\right)}^{2}+{\left(Z_{t}-Z_{s}\right)}^{2}}=\left(t_{f0}+t_{delay}+\frac{x}{f_{s}}\right)\times c\\ f_{D}=-\frac{2}{\lambda R}\left(R_{s}-R_{t}\right)\times \left(V_{s}-V_{t}\right)\\ \frac{X_{t}^{2}+Y_{t}^{2}}{R_{e}^{2}}+\frac{Z_{t}^{2}}{R_{p}^{2}}=1 \end{array}\right.$$

where *R_s_* = [*X_s_ Y_x_ Z_s_*]^T^ and *V_s_* are the orbit vector data of the SAR satellite in WGS84; *R_t_* = [*X_t_ Y_t_ Z_t_*]^T^ and *V_t_* are the position vector and velocity vector data of the target point in WGS84; *f_D_* is the Doppler centroid frequency, which can be calculated according to Equation (1); λ is the radar wavelength; *R* is the slant range; *X* is the column number of the target point in the SAR image; *c* is the speed of light; $R_{e}$ is the mean equatorial radius; and $R_{p}=\left(1-1/f\right)R_{e}$ is the polar radius with a flattening factor of f=298.255.

The track vector data recorded by the satellite are collected at equal time intervals. In the calculation, the orbit vector data of the SAR satellite should be fit by a polynomial and interpolated according to the corresponding azimuth time [14,15,16,17]. The azimuth time is the imaging moment of the control point corresponding to the line of an SAR image, and has been compensated according to the Equation (3). The image coordinates of the GCPs are obtained from the corner reflector points and the central points of the cross road in the SAR image, and are corrected for the impact of solid earth tides (SET), which are calculated using the International Earth Rotation Service (IERS) Conventions 2003 [18] and a small program called solid.exe [19].

**Figure 1:**
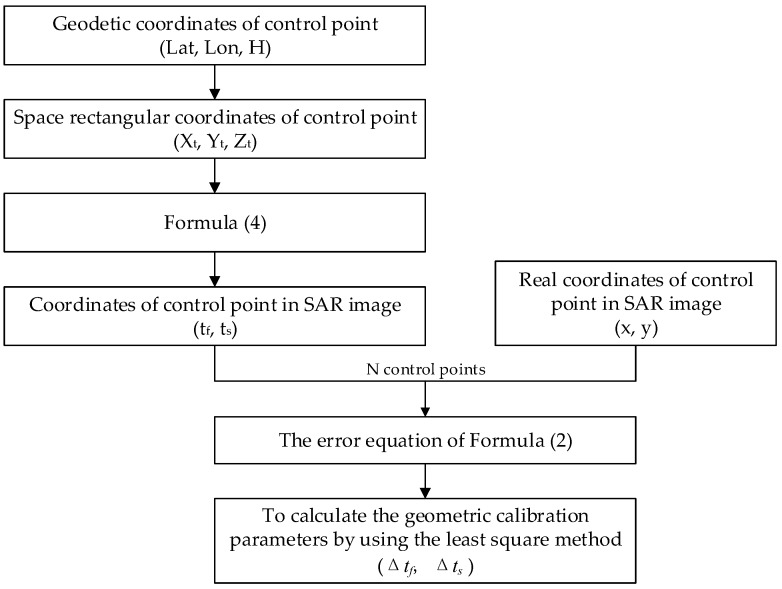
Flow chart of geometric calibration algorithm.

For the geometric calibration algorithm, based on the error equation of Equation (2), N control points are used to calculate the geometric calibration parameters by using the least square method, as shown in Figure 1. Then, the geometric calibration parameters are compensated to Equation (2). Based on the updated Equation (2), the geometric positioning accuracy after calibration is evaluated by Equation (4). The geometric positioning accuracy, which can be evaluated from an image coordinate system, can also be evaluated from the object coordinate system. For the image coordinate system, the geometric positioning accuracy can be evaluated by

(5) $$\{\begin{array}{c} \Delta x=x^{\prime }-x\\ \Delta y=y^{\prime }-y \end{array}$$

where Δx and Δy are difference value in range direction and azimuth direction; $x^{\prime }$ and $y^{\prime }$ are the pixel values of a control point calculated by Equation (4) according to (lat,lon,H) of the control point; and x and y are the pixel values of a control point in an SAR image. For the object coordinate system, the geometric positioning accuracy can be evaluated by

(6) $$\{\begin{array}{c} \Delta lat=lat^{\prime }-lat\\ \Delta lon=lon^{\prime }-lon \end{array}$$

where Δlat and Δlon are difference value in range direction and azimuth direction; $lat^{\prime }$ and $lon^{\prime }$ are the geographic coordinates of the control point calculated by Equation (4) according to (x,y,H) of the control point; and lat and lon are the real geographic coordinates of the control point. In any case, if there are a number of control points in one SAR image, the root-mean-square error (RMSE) is used as the evaluation index of geometric positioning accuracy.

For the geometric calibration and accuracy verification of the GF-3 satellite, we used a multimode hybrid geometric calibration method for spaceborne SAR, considering the atmospheric propagation delay. A detailed description of the method can be found in [11].

## 5. Results and Discussion

### 5.1. Experimental Data

**Figure 2:**
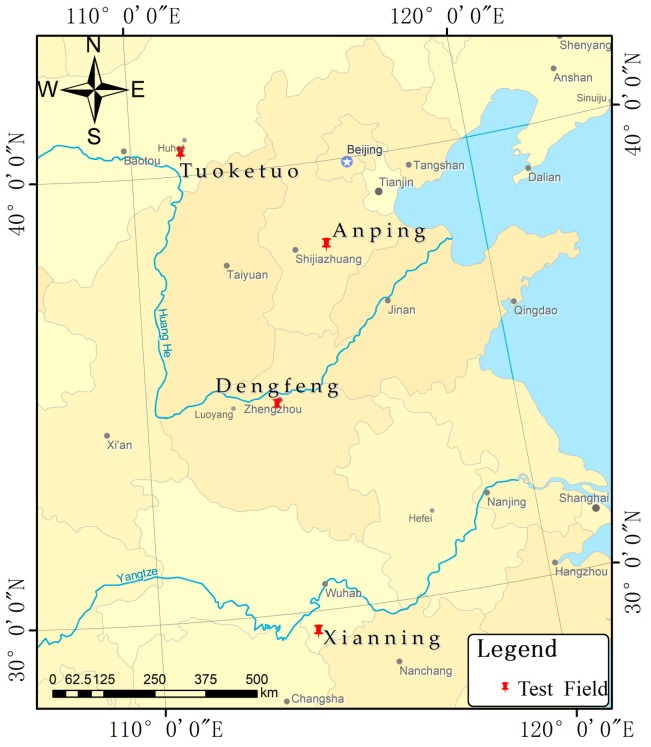
Regional distribution diagram of the ground control points (GCP)s.

In this study, GF-3 satellite images of 5 m resolution (Fine-Strip-1) and 8 m resolution (Full-Polarization-Strip-1) were used as the experimental data, and the acquisition times were from 11 January 2017 to 10 June 2017. To fully verify the improvement in geometric positioning accuracy for the GF-3 satellite, GF-3 SAR image data and ground control data of Anping County in Hebei Province, Tuoketuo country in the Nei Monggol Autonomous Region, Dengfeng City in Henan Province, and Xianning city in Hubei province were used. The GF-3 SAR image data of the study area is shown in Table 2. A regional distribution diagram of the GCPs is shown in Figure 2.

The automatic corner reflector equipment of the Songshan Remote Sensing Satellite Calibration Field in China were used as GCPs for Dengfeng city. GCPs for other areas were selected based on flat terrain and distinct features, and mainly involved the central points of cross roads and the corners of ponds. GCPs data were obtained from several Global Navigation Satellite System (GNSS) receivers, which can achieve centimeter-level positioning accuracy through static observation and real-time kinematic (RTK) technology.

### 5.2. Experimental Results and Analysis

**Figure 3:**
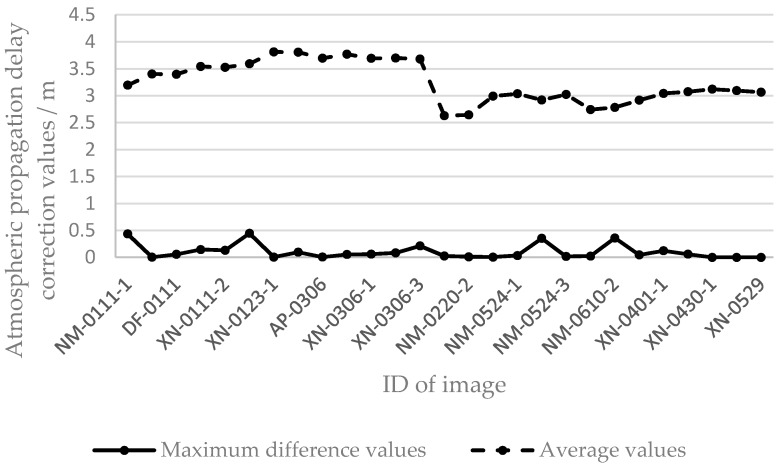
Variation in correction values in the range direction.

Based on external data from NCEP and CODE, the atmospheric propagation delay correction values for all GCPs in every SAR image of the GF-3 satellite were calculated, and the average and maximum difference values are shown in Figure 3.

In Figure 3, the maximum difference value represents the difference between the maximum and the minimum atmospheric propagation delay correction value in one scene image of the GF-3 satellite. These values indicate that the atmospheric propagation delay correction values of GCPs with a different spatial distribution were different. The largest maximum difference value was 0.446 m. Calculating the delay value of each point improved the positioning accuracy of a scene image by nearly 0.5 m. The average value represents the average atmospheric propagation delay correction value calculated by all GCPs in one scene image of the GF-3 satellite. The variation in average values indicates that the atmospheric propagation delay correction values at different times and regions were different. The maximum average value was 1.184 m. Calculating the delay value of each scene image improved the positioning accuracy by approximately 1 m. In summary, the positioning accuracy can be effectively improved by computing the atmospheric propagation delay correction value for each GCP and each scene. Also, the propagation delay (or electromagnetic bias) already has been computed and investigated to achieve higher position accuracy using GNSS-R systems [20,21].

The geometric calibration parameters of each GF-3 SAR image were calculated using the geometric calibration method described above, i.e., the initial range correction value and the initial azimuth time correction value. Then, the geometric calibration parameters were corrected for each image using the auxiliary parameters of that image. Finally, GCPs were used to verify the geometric positioning accuracy of the GF-3 satellite images. The inherent system errors were thus eliminated from the geometric positioning accuracy, as shown in Table 3. The residual errors mainly included the GCP selection error, SAR image distortion error, orbit position error, atmospheric propagation delay correction model error, SAR system delay random error, and antenna dispersion error. In one scene image, due to the short time of the aperture synthesis, the fixed system errors (including the orbit position error, atmospheric propagation delay correction model error, SAR system delay random error, and antenna dispersion error) were essentially eliminated, although a small amount of random error may have remained. In addition, because the GCPs contained accurate elevation information, the overlap and perspective contraction of the SAR images had little influence on the geometric positioning for GCPs. Therefore, the selection error of GCPs was the major residual error.

**Figure 4:**
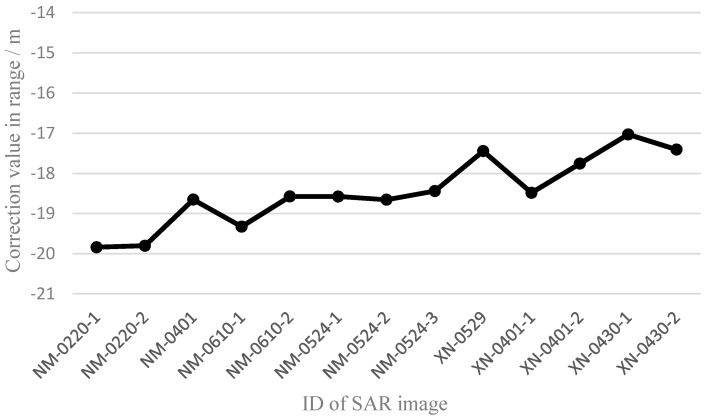
Variation trend of correction values in the range direction.

**Figure 5:**
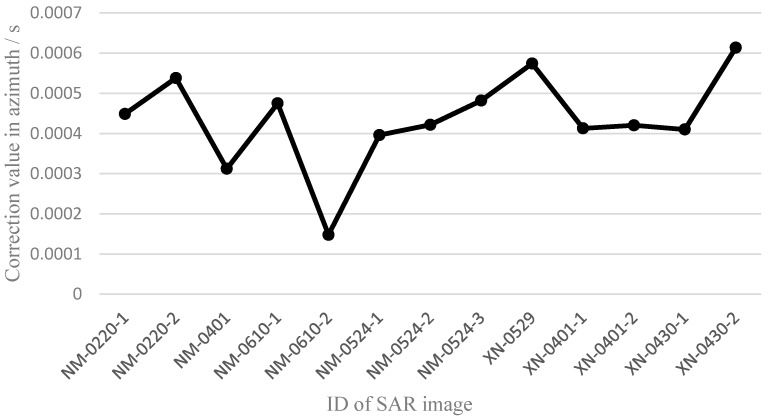
Variation trend of correction values in the azimuth direction.

Owing to the automatic corner reflectors used as GCPs in the Dengfeng region, the geometric positioning accuracy after single scene calibration was relatively high; up to 0.074 m. For the other regions, typical ground features were selected as GCPs. Because of the influence of speckle noise, image resolution, and signal-to-noise ratio, there will be more error in GCP selection using typical ground features. However, Table 3 shows that the geometric positioning accuracy was still better than 0.5 pixels. Because they only involved one GCP, the results of the last three images in the Xianning area were all zero pixels. Therefore, the selection accuracy of GCPs was better than 0.5 pixels in the experiments. Using the nine processing results of the GF-3 satellite that had the same pulse-width and bandwidth (24.99 μs and 40 MHz), the system errors were analyzed, as shown in Figure 4 and Figure 5.

The maximum difference of the slant range correction value was 2.811 m, and the root mean square error (RMSE) was 0.843 m (Figure 4). Because the pixel spacing in the range direction was approximately 2.25 m, the maximum difference of the slant range correction value was 1.03 pixels at the pixel scale. The maximum difference of the azimuth time correction value was 0.000465751 s, and the root mean square error (RMSE) was 0.000113152 s (Figure 5). Because the equivalent PRF in the azimuth direction was approximately 1216 Hz, the maximum difference of the azimuth time correction value was 0.52 pixels at the pixel scale. As a result, the trend of the two correction values was relatively stable. Thus, there were still some inherent system errors in the SAR images of the GF-3 satellite.

According to experimental data of the GF-3 satellite, four groups of geometric calibration experiments were performed. The experimental results of the GF-3 satellite geometric calibration are shown in Table 4.

(1) For a pulse-width and bandwidth of 24.99 μs and 50 MHz, and using Dengfeng city SAR image data as the calibration data, the system errors solved by the calibration data were corrected for the GF-3 satellite SAR image data in Tuoketuo country.
(2) For a pulse-width and bandwidth of 30 μs and 50 MHz, and using Tuoketuo country SAR image data as the calibration data, the system errors solved by the calibration data were corrected for GF-3 satellite SAR image data in Xianning city.
(3) For a pulse-width and bandwidth of 24.99 μs and 30 MHz, and using Xianning city SAR image data as the calibration data, the system errors solved by the calibration data were corrected for GF-3 satellite SAR image data in Anping country.
(4) For a pulse-width and bandwidth of 24.99 μs and 40 MHz, and using Xianning city SAR image data as the calibration data, the system errors solved by the calibration data were corrected for GF-3 satellite SAR image data in Tuoketuo country and Dengfeng city.

Table 2 shows that the maximum geometric positioning error of the FSM_I mode and QPSM_I mode were 2.781 m and 2.632 m after calibration, respectively. After geometric calibration, the geometric positioning accuracy of different times and regions was between 1.061 m and 2.781 m, and was relatively stable. This indicates that the experimental results were reliable. Moreover, the imaging time span of the experimental data was 5 months, from 11 January 2017 to 10 June 2017, further indicating that the geometric positioning performance of the GF-3 satellite SAR system was relatively stable. In summary, the geometric positioning accuracy of the GF-3 satellite was improved to better than 3 m.

## 6. Conclusions

The GF-3 satellite is the first multi-polarization SAR imaging satellite in China, having completed the in-orbit commissioning phase. Through an analysis of the geometric positioning accuracy of the GF-3 satellite, we have shown that some fixed system errors remain. Using a multimode hybrid geometric calibration method for spaceborne SAR, considering the atmospheric propagation delay, the SAR system of the GF-3 satellite was calibrated using GCPs and external atmospheric parameters. Moreover, the geometric positioning accuracy of the GF-3 satellite was evaluated using ground control data from several regions. The experimental results showed that the geometric positioning accuracy can be improved to better than 3 m.

## Figures and Tables

**Table 1:** Imaging modes of the GF-3 satellite.

Imaging Mode	Incidence Angle (°)	Resolution (m)	Imaging Width (km)	Polarization Mode
Spotlight (SL)	20–50	1	10	Selective single-polarization
Ultra-fine stripmap (UFSM)	20–50	3	30	Selective single-polarization
Fine stripmap 1 (FSM_I)	19–50	5	50	Selective dual-polarization
Fine stripmap 2 (FSM_II)	19–50	10	100	Selective dual-polarization
Standard stripmap (SSM)	17–50	25	130	Selective dual-polarization
Narrow scan SAR (NSC)	17–50	50	300	Selective dual-polarization
Wide scan SAR (WSC)	17–50	100	500	Selective dual-polarization
Global observation mode (GLO)	17–53	500	650	Selective dual-polarization
Full polarization stripmap 1 (FPSM_I)	20–41	8	30	Full polarization
Full polarization stripmap 2 (FPSM_II)	20–38	25	40	Full polarization
Wave mode (WAV)	20–41	10	5	Full polarization
Extended mode (EXT)	10–20	25	130	Selective dual-polarization
	50–60	25	80	Selective dual-polarization

SAR, synthetic aperture radar.

**Table 2:** GF-3 SAR image data of the study area.

Imaging Mode	Pulse-Width and Bandwidth	Date of Imaging	Imaging Region	Number of Images	ID of Image	Number of GCPs
FSM_I (5 m resolution)	24.99 μs and 50 MHz	11 January 2017	Tuoketuo country	2	NM-0111-1	8
					NM-0111-2	2
		11 January 2017	Dengfeng City	1	DF-0111	3
		11 January 2017	Xianning city	2	XN-0111-1	14
					XN-0111-2	7
	30 μs and 50 MHz	23 January 2017	Tuoketuo country	1	NM-0123	5
		23 January 2017	Xianning city	2	XN-0123-1	2
					XN-0123-2	11
FPSM_I (8 m resolution)	24.99 μs and 30 MHz	6 March 2017	Anping County	1	AP-0306	2
		10 March 2017	Xianning city	1	XN-0310	3
		6 March 2017	Xianning city	3	XN-0306-1	5
					XN-0306-2	8
					XN-0306-3	6
	24.99 μs and 40 MHz	20 February 2017	Tuoketuo country	2	NM-0220-1	4
					NM-0220-2	2
		1 April 2017	Tuoketuo country	1	NM-0401	3
		24 May 2017	Tuoketuo country	3	NM-0524-1	3
					NM-0524-2	11
					NM-0524-3	3
		10 June 2017	Tuoketuo country	2	NM-0610-1	5
					NM-0610-2	6
		1 April 2017	Dengfeng City	1	DF-0401	2
		1 April 2017	Xianning city	2	XN-0401-1	7
					XN-0401-2	4
		30 April 2017	Xianning city	2	XN-0430-1	1
					XN-0430-2	1
		29 May 2017	Xianning city	1	XN-0529	1

**Table 3:** Verification of geometric accuracy after single scene calibration.

Imaging Mode	Pulse-Width and Bandwidth	ID of Image	Line (Pixel)	Sample (Pixel)	2-D (Pixel)
FSM_I (5 m resolution)	24.99 μs and 50 MHz	NM-0111-1	0.122	0.308	0.331
		NM-0111-2	0.091	0.179	0.201
		DF-0111	0.014	0.168	0.169
		XN-0111-1	0.121	0.353	0.373
		XN-0111-2	0.07	0.332	0.339
	30 μs and 50 MHz	NM-0123	0.114	0.17	0.205
		XN-0123-1	0.052	0.216	0.222
		XN-0123-2	0.189	0.437	0.476
FPSM_I (8 m resolution)	24.99 μs and 30 MHz	AP-0306	0.118	0.071	0.138
		XN-0310	0.079	0.359	0.367
		XN-0306-1	0.116	0.346	0.365
		XN-0306-2	0.15	0.192	0.243
		XN-0306-3	0.163	0.221	0.275
	24.99 μs and 40 MHz	NM-0220-1	0.064	0.253	0.261
		NM-0220-2	0.175	0.013	0.176
		NM-0401	0.343	0.102	0.358
		NM-0524-1	0.025	0.156	0.157
		NM-0524-2	0.086	0.233	0.249
		NM-0524-3	0.115	0.103	0.155
		NM-0610-1	0.086	0.24	0.255
		NM-0610-2	0.14	0.21	0.253
		DF-0401	0.073	0.008	0.074
		XN-0401-1	0.118	0.186	0.22
		XN-0401-2	0.144	0.36	0.388
		XN-0430-1	0	0	0
		XN-0430-2	0	0	0
		XN-0529	0	0	0

**Table 4:** Comparison of geometric positioning accuracy before and after correcting for geometric calibration parameters.

Imaging Mode	Pulse-Width and Bandwidth	ID of Image	Geometric Calibration	Azimuth (Pixel)	Range (Pixel)	2-D	
						(Pixel)	(m)
FSM_I (5 m resolution)	24.99 μs and 50 MHz	NM-0111-1	Before	0.463	9.679	9.690	21.802
			after	0.176	1.217	1.230	2.781
		NM-0111-2	Before	0.528	9.840	9.854	22.175
			after	0.106	1.121	1.126	2.537
	30 μs and 50 MHz	XN-0123-1	Before	0.432	10.452	10.461	23.540
			after	0.301	0.505	0.588	1.481
		XN-0123-2	Before	0.525	9.940	9.954	22.412
			after	0.301	0.441	0.534	1.374
QPSM_I (8 m resolution)	24.99 μs and 30 MHz	AP-0306	Before	0.655	5.211	5.252	23.663
			after	0.136	0.264	0.296	1.366
	24.99 μs and 40 MHz	NM-0220-1	Before	0.549	9.997	10.013	22.686
			after	0.079	1.018	1.021	2.330
		NM-0220-2	Before	0.677	9.983	10.007	22.763
			after	0.186	0.969	0.986	2.413
		NM-0401	Before	0.531	9.628	9.643	21.824
			after	0.410	0.469	0.623	2.379
		NM-0524-1	Before	0.482	9.613	9.625	21.778
			after	0.112	0.451	0.465	1.190
		NM-0524-2	Before	0.520	9.599	9.613	21.774
			after	0.116	0.515	0.528	1.324
		NM-0524-3	Before	0.597	9.546	9.564	21.717
			after	0.115	0.377	0.394	1.061
		NM-0610-1	Before	0.584	9.817	9.834	22.208
			after	0.088	0.794	0.799	1.850
		NM-0610-2	Before	0.228	9.507	9.510	21.413
			after	0.435	0.473	0.642	2.632
